# Strong population structure in a species manipulated by humans since the Neolithic: the European fallow deer (*Dama dama dama*)

**DOI:** 10.1038/hdy.2017.11

**Published:** 2017-03-29

**Authors:** K H Baker, H W I Gray, V Ramovs, D Mertzanidou, Ç Akın Pekşen, C C Bilgin, N Sykes, A R Hoelzel

**Affiliations:** 1 School of Biological and Biomedical Sciences, Durham University, Durham, UK; 2 Department of Biology, University of Athens, Athens, Greece; 3 Department of Biology, Middle East Technical University, Ankara, Turkey; 4 Department of Molecular Biology and Genetics, Yüzüncü Yıl University, Van, Turkey; 5 Department of Archaeology, University of Nottingham, Nottinghamshire, UK

## Abstract

Species that have been translocated and otherwise manipulated by humans may show patterns of population structure that reflect those interactions. At the same time, natural processes shape populations, including behavioural characteristics like dispersal potential and breeding system. In Europe, a key factor is the geography and history of climate change through the Pleistocene. During glacial maxima throughout that period, species in Europe with temperate distributions were forced south, becoming distributed among the isolated peninsulas represented by Anatolia, Italy and Iberia. Understanding modern patterns of diversity depends on understanding these historical population dynamics. Traditionally, European fallow deer (*Dama dama dama*) are thought to have been restricted to refugia in Anatolia and possibly Sicily and the Balkans. However, the distribution of this species was also greatly influenced by human-mediated translocations. We focus on fallow deer to better understand the relative influence of these natural and anthropogenic processes. We compared modern fallow deer putative populations across a broad geographic range using microsatellite and mitochondrial DNA loci. The results revealed highly insular populations, depauperate of genetic variation and significantly differentiated from each other. This is consistent with the expectations of drift acting on populations founded by small numbers of individuals, and reflects known founder populations in the north. However, there was also evidence for differentiation among (but not within) physically isolated regions in the south, including Iberia. In those regions we find evidence for a stronger influence from natural processes than may be expected for a species with such strong, known anthropogenic influence.

## Introduction

The evolution of biodiversity in natural systems is driven by various factors, both environmental and biotic (see Benton, 2009). Environmental factors can include the changing distribution of resources, vicariance events separating populations into allopatry and displacement into refugia. In Europe, glacial periods are known to have driven a diversity of species into three primary southern refugia located in Iberia, Italy and the Balkans from where they later recolonised central and northern Europe through range expansions (see Hewitt, 2000). For most species there are anthropogenic factors superimposed onto this process at varying levels, and during different time frames (see, for example, Goudie, 2013). By the time we reach the modern era, many species’ distributions will have been affected by human-induced habitat fragmentation, exploitation and translocation. Comparing affected populations allows us to test the hypotheses about the more general mechanisms and processes for the evolution of intraspecific diversity, and to draw inference about effective conservation strategies. In this study we investigate the relative contributions of anthropogenic and natural processes for the European fallow deer (*Dama dama dama*), a species that has been integrated into human culture since the Neolithic (Yannouli and Trantalidou, 1999).

Although represented in many northern archaeological faunal assemblages dating to before the last glacial period (~110–12 Ka), fallow deer became extinct across north-west Europe during the last glacial period, surviving only in refugia in southern Europe (see, for example, Arslangundogdu *et al.*, 2010). There is evidence to suggest that Anatolia constituted one such refugia for fallow deer (Masseti, 1996; Chapman and Chapman, 1997) but the extent to which other areas also provided refuge remains unknown. It has been suggested that additional refugia may have existed in Sicily and the southern Balkans (Masseti, 1996, 1999). There is no clear archaeological evidence of early colonisation of the Iberian Peninsula (Davis and MacKinnon, 2009), suggesting an exception to the usual three main refugia (cf., Hewitt, 2000). Unlike many other temperate species, fallow deer did not naturally recolonise central and northern Europe after the last Ice Age. Pemberton and Smith (1985) suggested that this is because reduced genetic variation retarded their ability to adapt to altered environments, or local adaptation reduced their ability to survive in the north. This lack of expansion from southern refugia is atypical, but not unique (see Bilton *et al.*, 1998).

Instead of natural recolonisation, the Holocene diffusion of the European fallow deer from refugial populations appears to have been initiated by humans (see Chapman and Chapman, 1980). Consequently, it is often considered that among cervids, the current distribution of fallow deer is the most anthropogenically influenced (Putman, 1988; Chapman and Chapman, 1997). Archaeozoological evidence suggests that movement of fallow deer began early in the Neolithic within continental and insular Greece (Yannouli and Trantalidou, 1999) and the Aegean islands of Crete (Harris, 2015) and Rhodes (Masseti *et al.*, 2008). Translocations increased during the Bronze Age, when this species also began its diffusion into the Western Mediterranean (Yannouli and Trantalidou, 1999). The Romans introduced small numbers of fallow deer into central and northern Europe and into Portugal (Davis and MacKinnon, 2009), Switzerland (Schmid, 1965), France (Lepetz and Yvinec, 2002) the Netherlands (Prummel, 1975), Austria and Britain (Sykes, 2004), even maintaining it locally in some cases (Sykes *et al.*, 2006, 2011; Madgwick *et al.*, 2013). With the withdrawal of the Roman Empire, breeding populations in at least Northern Europe likely perished with later medieval introductions giving rise to many populations today (Sykes *et al.*, 2016). More recently, during the nineteenth and twentieth centuries, humans went on to spread this species across the globe into the Near East, North and South America, northern and southern Africa, Australia, New Zealand and widely across Europe (Chapman and Chapman, 1980).

Although fallow deer have prospered within much of their introduced range, numbers within its postglacial native range of Turkey have dwindled because of uncontrolled hunting (Chapman and Chapman, 1997). Currently, there are three populations in Turkey that collectively number no more than 200 individuals. Elsewhere, the oldest populations in the Balkans and Sicily have been long extirpated (during the Medieval period), although more recent reintroduction events in the past 50 years have re-established populations in parts of the Balkans, such as in Bulgaria. A population on Rhodes is morphologically and genetically differentiated (based on mitochondrial DNA (mtDNA)), suggesting a founder population introduced by humans (Masseti *et al.*, 2008).

Previous genetic studies on fallow deer have generally shown low levels of variation within putative populations (Pemberton and Smith, 1985; Hartl *et al.*, 1986; Randi and Apollonio, 1988; but see also; Masseti *et al.*, 1997), said to result from genetic drift, founder effects and/or bottlenecks. The most extensive mtDNA study to date was by Ludwig *et al.* (2012) who examined 365 fallow deer samples for the mtDNA control region (400 bp) encompassing 22 locations in Germany. They identified three distinct regional clusters, and although there was some indication the German founders were of Turkish origin, the pattern was complex. Other studies have investigated microsatellite DNA variation, but only at local scales (see, for example, Say *et al.*, 2003 in Ireland and Webley *et al.*, 2007 in Australia). Our study is the first to investigate fallow deer diversity across most of the species geographic range.

Here we investigate the relative influence of natural and anthropogenic processes on the evolution of genetic diversity. Using the comparative analysis of mtDNA HVR1 (hypervariable region 1) sequence data and 10 microsatellite DNA loci, we test the following hypotheses: (1) that a signature reflecting postglacial refugia can still be detected; (2) that the timing and pattern of population divisions largely reflect the historical data on known patterns of translocation; and (3) that populations in the north reflect an amalgamation of founder events reflecting human translocations from the southern refugial species range.

## Materials and methods

### Sampling and DNA extraction

**Figure 1:**
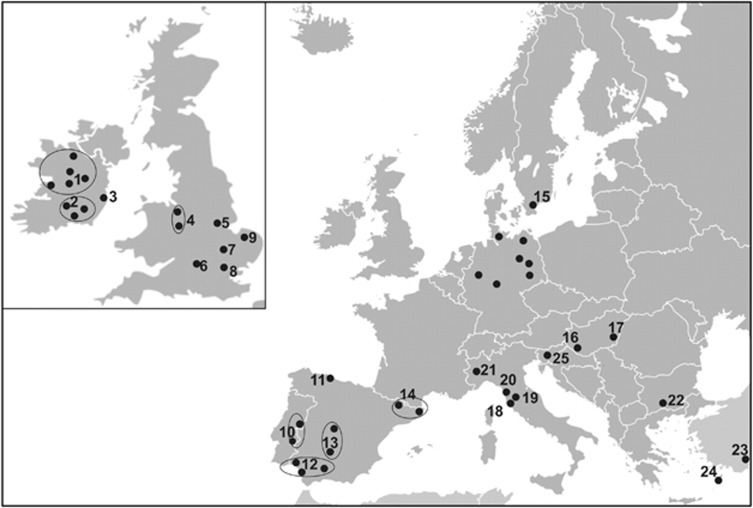
Map showing fallow deer sampling locations from across Europe. Locations are individually numbered to correspond with the samples presented in Table 1. Locations for samples representing each of the unique haplotypes found in Germany are detailed in Ludwig *et al.* (2012).

Samples were collected from 364 fallow deer across 10 European countries and Canada (Figure 1 and Table 1). This included samples from five populations in Iberia (well distributed across Spain and Portugal; Figure 1), three in Italy (clustered near Florence) and one in Anatolia (Turkey; Table 1). Tissue samples, mostly tongue or ear clippings (1 cm^3^), were stored in 20% dimethylsulphoxide saturated with NaCl (Amos and Hoelzel, 1991). DNA was extracted using a phenol chloroform method (Sambrook *et al.*, 1989). Bone samples were crushed and 50–100 mg of powder was left to incubate in 500 μl of digestion buffer (1% sodium dodecyl sulphate, 1 mM EDTA and 1 M Tris) supplemented with proteinase K (20 mg ml^−1^). DNA was extracted from the digested solution using a Qiagen QIAquick PCR purification kit (Qiagen, Hilden, Germany) and stored at −20 °C. DNA was isolated from blood samples using a salting-out method (after Lahiri and Schnabel, 1993).

### Genotyping

Individuals (*N*=364) were genotyped using 10 previously published microsatellites shown to be polymorphic in fallow deer (see Supplementary Table S1 for details and citations). The software MicroChecker v. 2.2.3 (Van Oosterhout *et al.*, 2004) was used to test microsatellite loci for null alleles, large allele dropout and scoring errors due to stutter peaks. Tests for deviation from Hardy–Weinberg equilibrium (HWE) for each locus–population combination were carried out using exact tests implemented in ARLEQUIN v. 3.5 (Excoffier and Lischer, 2010). Tests for linkage disequilibrium were carried out for each pair of loci using an exact test based on a Markov chain method as implemented in Genepop v. 3.4 (Raymond and Rousset, 1995).

### Mitochondrial D-loop sequencing

A mtDNA control region fragment of 683 bp was amplified using two primers developed by Randi *et al.* (1998): Lcap Pro 5′-CGTCAGTCTCACCATCAACCCCCAAAG-3′ and Hcap Phe 5′-GGGAGACTCATCTAGGCATTTTCAGTG-3′. PCR reactions (20 μl total volume) contained 0.2 μM each primer, 0.2 mM each dNTP, 10 mM Tris-HCL pH 9.0, 1.5 Mm MgCl_2_ and 0.4 units of *Taq* polymerase (New England Biolabs, Ipswich, MA, USA) with cycle conditions: 95 °C for 5 min; 35 cycles at 94 °C for 45 s, 51 °C for 45 s and 72 °C for 45 s; and 72 °C for 5 min. PCR products were purified using Qiagen PCR purification columns (Qiagen, Inc.) and directly sequenced using an ABI 377 automated sequencer (Foster City, CA, USA). A representative subset of the available samples (*N*=203) were sequenced.

### Genetic diversity and structure

The mtDNA sequences were aligned manually using Geneious v. R6 (Biomatters, Auckland, New Zealand). The program DnaSP v. 10.4.9 (Rozas *et al.*, 2003) was used to calculate mtDNA polymorphism estimated as haplotypic diversity (*hd*; Nei and Tajima, 1981), nucleotide diversity (π, Nei, 1987) and average pairwise nucleotide divergence (*k*). Locations with <10 samples were excluded from diversity and *F*_ST_ analyses, as was Germany where only samples representing each of the unique haplotypes from the former study of Ludwig *et al.* (2012) were used. The relationship among haplotypes (including those originating from Germany) was examined by constructing median-joining networks (Bandelt *et al.*, 1999) as implemented in the program NETWORK v. 3.1.1.1 (fluxus-engineering.com). To assess the level of genetic differentiation between pairs of populations, *F*_ST_ (after Weir and Cockerham, 1984) was calculated for mtDNA and microsatellite DNA loci using ARLEQUIN v. 3.5 (Excoffier and Lischer, 2010). Significance was tested using 1000 permutations with Bonferroni correction applied.

For microsatellite DNA data, allelic richness for each locus and population and *F*_IS_ were calculated using the program FSTAT v. 2.9.3 (Goudet, 2001). The sequential Bonferroni method was used to correct for type I errors (Rice, 1989). The program STRUCTURE v. 2.0 (Pritchard *et al.*, 2000) was used to infer the putative number of populations (*K*) based on microsatellite data. This was assessed using Δ*K*, a measure of the second-order rate of change in the likelihood of *K* (Evanno *et al.*, 2005) providing the highest hierarchical level of structure, and by comparing the posterior probabilities for the values of *K* with the highest Ln *P*(*X*|*K*). Five independent runs were performed for each value of *K* (1–15) at 10^**6**^ Markov chain Monte Carlo repetitions and 10^**5**^ burn-in using no prior information on sampling population and applying the correlated allele frequency and admixture ancestry models. Population structure not detected at the higher hierarchical level, but apparent in other analyses, was explored further by re-running the program with subsets of population samples.

Patterns of microsatellite differentiation were visualised using a factorial correspondence analysis implemented in GENETIX v. 4.0 (Belkhir *et al.*, 2000; see She *et al.*, 1987) and using a principal coordinate analysis implemented in GenAlEx 6.5 (Peakall and Smouse, 2012). Isolation by distance (based on microsatellite DNA loci) was assessed with a Mantel test (10 000 permutations) using Genepop v. 4.2 (Raymond and Rousset, 1995).

### Estimates of gene flow

Long-term average gene flow between southern populations (Iberia: Spain and Portugal, Italy and Turkey), across 10 microsatellite loci, was investigated using coalescent analyses in Migrate-n v. 3.6.11 (Beerli and Felsenstein, 2001; Beerli, 2006, 2009). The Bayesian approach was implemented and a Brownian-motion model for microsatellite data applied. Broad, uniform priors were set for θ (min=0, max=100, delta=10) and 4 Nm (θ M) (min=0, max=100, delta=10). Delta was set at 1/10 maximum as recommended by authors (see Beerli, 2009). Runs were performed with 20 replicates and a static heating scheme using four chains. A burn-in of 40 000 trees was applied and 40 000 genealogies were recorded with a sampling increment of 100. Convergence was assessed through examination of effective sample size values (>1000) and posterior probability histograms for smooth, single-peak distributions. Multiple runs were also performed to confirm that posterior probabilities of parameters were converging on similar estimates.

BayesAss v.1.3 (Wilson and Rannala, 2003) was used to investigate recent gene flow between southern populations (as above) and among all sampled populations using 10 microsatellite loci. The burn-in length was set to 10^7^ followed by 10^8^ Markov chain Monte Carlo iterations with a sampling interval of 1000 iterations (chosen after testing alternatives and assessing convergence). Mixing parameters were adjusted to improve mixing for the southern populations data set (Δ*A*=0.3, Δ*F*=0.7 and Δ*M*=0.1) and the data set that included all populations (Δ*A*=0.4, Δ*F*=0.1 and Δ*M*=0.15). Trace files were viewed in Tracer v.1.6 (Rambaut *et al.*, 2014) and the log-probability was examined for convergence and good chain mixing. Analyses were run multiple times to check for convergence. Circos plots of migration dynamics were generated in R v. 3.0 (R Core Team, 2013) from the BayesAss outputs using the package *circlize* (Gu *et al.*, 2014), following Sander *et al.* (2014).

### Inferring alternative scenarios using ABC

**Figure 2:**
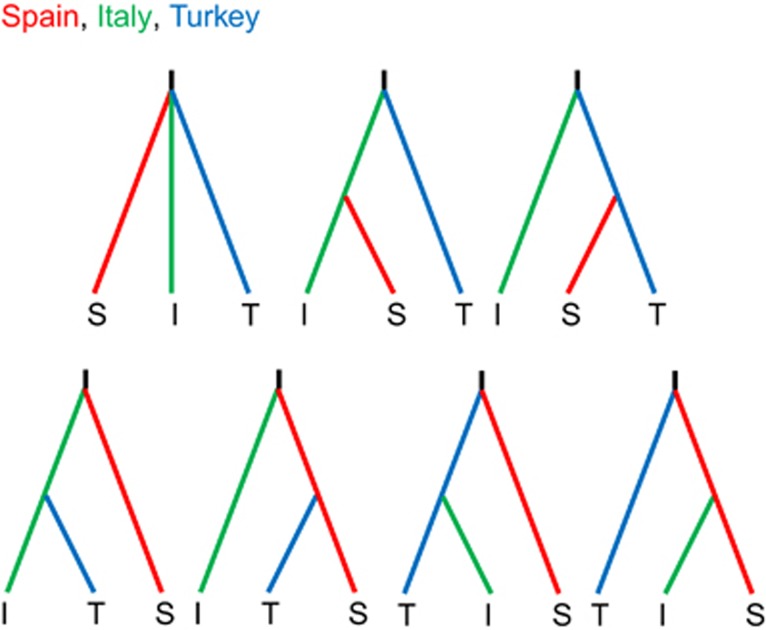
Graphical representation of the seven scenarios considered with approximate Bayesian computation using the software DIYABC. See Table 3 and Supplementary Table S7 and Supplementary Figures S4 and S5 for the results of the analysis.

Approximate Bayesian computation (ABC) analysis was run using the software package DIYABC (Cornuet *et al.*, 2008) to investigate different possible demographic scenarios. We restricted this analysis to the southern population samples representing putative Anatolian (*N*=24), Italian (*N*=21) and Iberian (*N*=26) populations, corresponding to the geographically isolated regions that supported refugial populations for various other species (see Hewett, 2000). We draw inference from microsatellite DNA alone, and also for microsatellite and mtDNA data combined. Including mtDNA has the potential to reduce error (Cornuet *et al.*, 2010), though perhaps not in this case as explained in the Discussion section. Simulations were run using broad, flat priors (10–10 000 for *N*_e_ and *t*, and 10–50 000 for Tb) and 1 000 000 data sets. Parameter estimates were assessed for the 6000 simulated data sets closest to the observed data using direct and logistic regressions. The ‘direct’ estimate is the number of times the scenario is found among data sets closest to the observed data set. The logistic regression compares the proportion of the scenario (dependent variable) with the differences between observed and simulated data set summary statistics (see Cornuet *et al.*, 2010). Broad, uniform priors for the mutation rate ranged from 1 × 10^−5^ to 1 × 10^−2^ with a mean value set at 5 × 10^−4^ applied to all loci (consistent with published estimates for microsatellite DNA loci; see, for example, Huang *et al.*, 2002). We compared seven scenarios (Figure 2) as follows: (1) all three populations diverged at the same time; (2) the Iberian population (represented by the population from Spain) was derived from Italy after Italy diverged from Anatolia (Turkey); (3) Spain diverged from Turkey after Turkey diverged from Italy; (4) Turkey diverged from Italy after Italy diverged from Spain; (5) Turkey diverged from Spain after Spain diverged from Italy; (6) Italy diverged from Turkey after Turkey diverged from Spain or (7) Italy diverged from Spain after Spain diverged from Turkey.

We do not precisely know the generation time, but fallow deer produce one offspring annually starting in their first or second year. If we assume that age of maturity (*α*) is 2 years, adult survival of females is 16 years (Chapman and Chapman, 1997) and the populations are stable (*λ*=1), then calculated generation time would be ~1 (see formula 2 in Gaillard *et al.*, 2005). However, a published generation time suggests 5.9 years (Lemaitre and Gaillard, 2012). Therefore, we present tabulated data assuming a generation time of 1 year, but discuss how the published value of 5.9 years would affect our estimates of splitting times.

## Results

### Diversity

Genetic diversity was low within putative populations for all microsatellite markers (Table 1). All marker loci were polymorphic across the whole sample but some were monomorphic within populations and showed fixed differences (see Supplementary Table S2). Significant deviation from HWE within southern population samples was rare after Bonferroni correction (see Table 1). The sample set from England showed the greatest degree of deviation from HWE (significant at 3 loci for heterozygote deficit) which could be because of the Wahlund effect (seen when multiple populations are grouped together as one; Wahlund, 1928). In England, this could be associated with admixture from multiple translocations (Table 1), but there was no similar effect for multiple populations within either Italy or Iberia (suggesting panmixia among locations within these regions). Other regions were either sampled for small numbers in multiple locations, or from just one location, and hence ‘populations’ are subsequently defined by country of origin. Significant linkage disequilibrium was detected between HAUT127 and BM4505. Analyses were conducted with and without one of these loci, but because differences between corresponding analyses were negligible (data not shown), statistics presented are those calculated across all loci.

### Population structure

**Figure 3:**
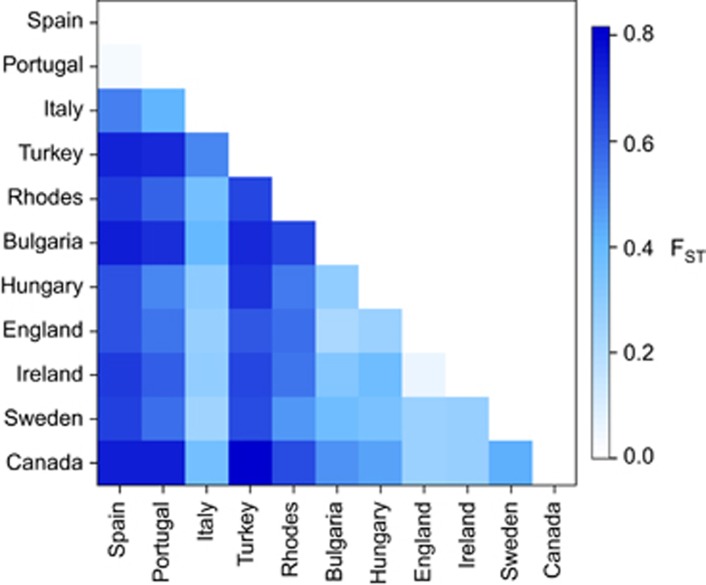
Heatmap of pairwise *F*_ST_ values estimated from microsatellite data between all populations. Darker shading indicates higher *F*_ST_ values, as indicated by key to the right of the figure.

**Figure 4:**
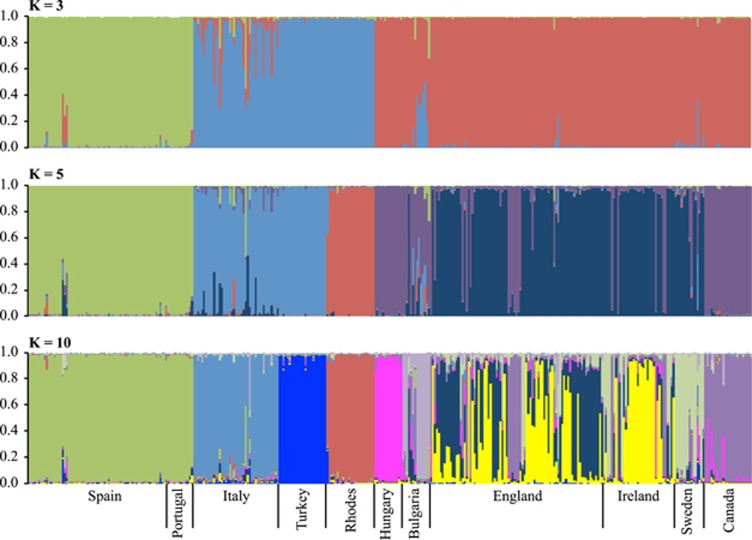
Assignment probabilities of individuals to putative population clusters at *K*=3, *K*=5 and *K*=10 using the program STRUCTURE 2.3.2. Locations where individuals were sampled are indicated below the graphs. Likelihood support values associated with these analyses are provided in Supplementary Figure S1.

**Figure 5:**
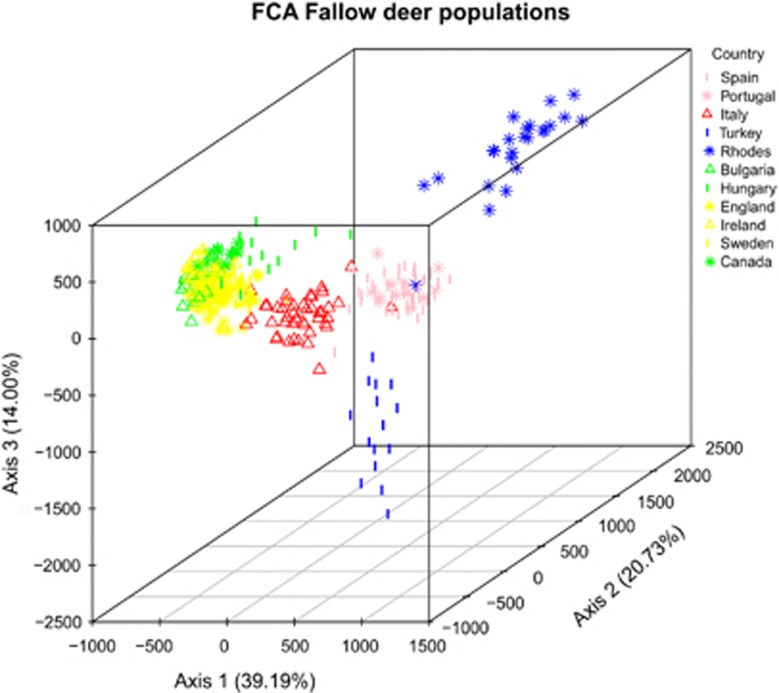
Factorial correspondence analysis (FCA) of population multilocus scores computed using GENETIX based on a comparison of the first three factors.

Differentiation among populations was strong, with all pairwise *F*_ST_ values large and significant (except for the comparisons between Spain and Portugal and Ireland and England; Figure 3 and Supplementary Table S3); however, there was a pattern to the magnitude of *F*_ST_ values. Comparisons between Turkey, Italy and Spain/Portugal had relatively high *F*_ST_ values, whereas *F*_ST_ values among northern populations and between Italy and the northern populations were lower (see Figure 3). For assignments in Structure the mean Ln*P*(*K*) values increased gradually with a maximum at *K*=10 (Figure 4 and Supplementary Figures S1). The gradual change in Ln*P*(*K*) meant that a useful value for delta *K* could not be determined (see Supplementary Figure S1). The three southern populations, Turkey, Italy and Iberia (Spain and Portugal combined), reflecting the typical refugial regions are distinguished after *K*=5. However, the northern populations (hereafter defined as England, Ireland, Sweden, Hungary, Bulgaria and Canada) show greater complexity as *K* increases (Figure 4). At *K*>5, Rhodes is clearly distinguished from Turkey. This is also evident in the factorial correspondence analysis (Figure 5) and principal coordinate analysis (Supplementary Figure S2) analyses. These ordination plots also show the clear distinction between Italy, Turkey and Iberia, together with an association between Italy and the northern populations and overlap among the northern populations. Both Structure and ordination plots show no strong evidence for substructure within Turkey, Italy or Iberia, but the Structure plots reveal complexity in the sample from England. When *K*=10, it is possible to track a connection between assignments from regions within the UK sample to each of Ireland, Sweden and Canada, consistent with known translocations (as described in more detail in the Discussion section), and some possible indication of admixture.

We generated long-term (Migrate-n) and short-term (BayesAss) estimates of gene flow, both among the southern populations on their own (comparing Iberia=Spain+Portugal, Italy and Turkey) and (with BayesAss) among all populations. The estimates of directional, long-term gene flow among these three southern populations (based on Migrate-n) indicate low levels of migration and no clear pattern of directionality (Supplementary Table S4). Theta estimates suggest that *N*_e_ is larger in Italy than for Iberia or Turkey, though confidence limits are broad and all are bounded by zero (Supplementary Table S4). BayesAss also suggested low levels of gene flow between these three populations, and a similar lack of directionality among populations, though there is some support for greater migration from Iberia to Turkey than from Turkey to Iberia (Supplementary Table S4 and Supplementary Figure S3a). When all populations were included, all showed high levels of insularity and no clear pattern of directional gene flow, with the exceptions of strong directional gene flow from Spain to Portugal and from England to Ireland (Supplementary Figure S3b).

**Figure 6:**
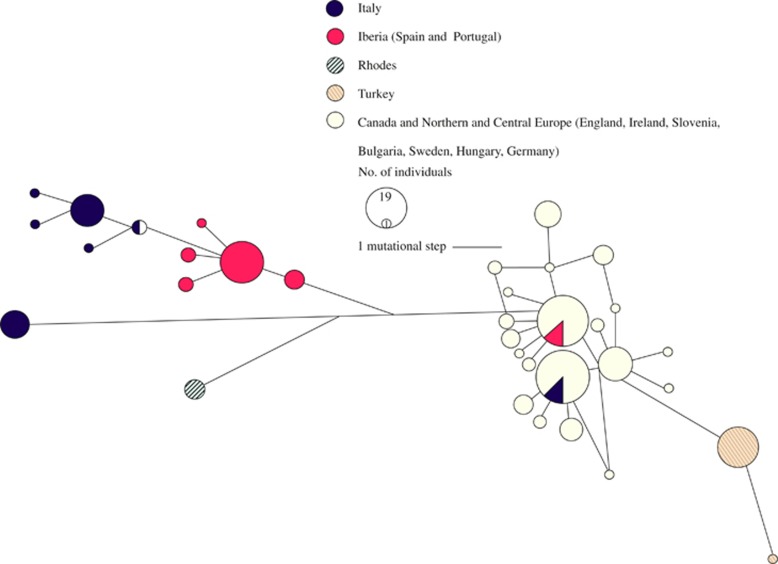
Median joining network of phylogenetic relationships among fallow deer mitochondrial haplotypes where the size of the circle indicates relative frequency of the haplotype. Haplotypes represented are based on 683 base pairs of the mtDNA control region.

The mtDNA network, based on 683 bp of control region sequence for 203 samples, shows clustering of the northern populations and separation between putative refugial populations, though there may be incomplete lineage sorting for the Italian and Iberian lineages, or introgression from the Iberian lineage into the Italian population (see Figure 6). Most populations show low levels of mtDNA diversity, with the exception of Italy, where both haplotype and gene diversity are relatively high (Table 2 and Supplementary Table S5). Pairwise *F*_ST_ values based on mtDNA sequence data reflect the pattern of haplotype clustering seen in the network, with highly significant differentiation between all but the northern populations that cluster together in the network (Figure 6 and Supplementary Table S6). Previously, Massetti *et al.* (2008) identified an 80 bp insertion unique to the Rhodian samples. We identify this same insertion for Rhodian samples as well as an additional 21 bp insertion found in some samples from Iberia and Italy but not in other countries.

### Population dynamics

Model testing was undertaken just for the three main southern populations (Turkey, Italy and Spain). These regions represent putative refugial populations in a wide range of species (see Hewitt, 2000). We used ABC analysis to test possible postglacial founder expansion scenarios (Figure 2). Our results strongly favour scenario one, the trifurcation (see direct and logistic regressions in Supplementary Figure S4), and the estimated time of division among the three populations is in the late Pleistocene or early Holocene (see Table 3). Inference from the logistic regression is often taken as more robust (see Cornuet *et al.*, 2010), but in our case results for the direct and logistic regression estimates are very similar. If the generation time was as high as 5.9 (Lemaitre and Gaillard, 2012), the splitting time estimate (from the microsatellite DNA data) would be 74 930 (10 915–224 200) years. For the analysis based only on microsatellite DNA, the logistic regression value for the trifurcation scenario is much higher (0.5833, 95% confidence interval (CI)=0.5605–0.6061) compared with the next highest scenario, scenario 7 where Italy splits from Spain (0.129, 95% CI=0.1138–0.1443). This is tighter when mtDNA is included, but there is still no overlap between the result for the trifurcation scenario (scenario 1) and the next best supported, in this case scenario 2 where Spain diverges from Italy (scenario 1: 0.2332, 95% CI=0.224–0.2424; scenario 2: 0.1958, 95% CI=0.1871–0.2046). A principle component analysis of the fit between posterior and observed data sets shows a good match for scenario 1 for both runs (microsatellite loci alone and mtDNA and microsatellites combined; Supplementary Figure S5). The match between parameter estimates and observed values associated with diversity and divergence is strong for scenario 1, especially for the microsatellite DNA only run (Supplementary Table S7). The posterior distributions for mutation rates were 2.1 × 10^−4^ (±s.d. 1.02 × 10^−4^) for microsatellite DNA loci and 2.93 × 10^−7^ (±s.d. 1.49 × 10^−7^) for the mtDNA sequence (per site per year). The *N*_e_ estimates from ABC again suggest that the Italian population is larger than the other two and the putative ancestral population (Table 3).

If we consider the less well supported scenario 2 (whereby the Spanish population diverges from the Italian population; time ‘*t*’), the posterior distribution for this scenario gives similar, overlapping posterior distributions as the older (time ‘Tb’) division, each near the start of the Holocene (*t*=5720 YBP, 95% CI=608–17 500; Tb=8160, 95% CI=713–26 600). It is possible for a relatively simple model (such as the ‘trifurcation’ model in scenario 1) to be supported over more complex models through a lack of power. However, the result from scenario 2 suggests that this may not the case for these data, as there is little support for resolving distinct time points for the *t* and Tb divisions in this scenario (the second best supported scenario by most measures, see Supplementary Figure S4).

## Discussion

### Divergence and diversity

The two most striking features of fallow deer population diversity in its European range are the very low levels of diversity within regional populations, and the high degree of differentiation between them (with *F*_ST_ values exceeding that reported among congeneric species; see, for example, Forbes *et al.*, 1995). At the mtDNA locus, both haplotype and gene diversity metrics were low for a given sampling region, consistent with other cervid populations that had been through population bottlenecks (see, for example, Haanes *et al.*, 2010; Rosvold *et al.*, 2012; Baker and Hoelzel, 2013), with the exception of Italy. This exception is consistent with the *N*_e_ estimates where Italy mean estimates are highest (compared with Spain and Turkey; Table 3), though the confidence limits are broad and overlapping. Microsatellite locus diversity is less readily compared among studies or species, as the choice of loci varies, and the mutation rates can vary among loci (see, for example, Chakraborty *et al.*, 1997). However, even in that context, these diversity values are low. Among our various sample populations *H*_e_ varied from 0.14 to 0.48, AR varied from 1.46 to 2.42 and there were 1–4 alleles among loci and populations (Table 1). Again, Italy was highest among these, and evidently not because of mixing populations as *F*_IS_ was only marginally positive and heterozygosity was not significantly lower than HWE expectations (Table 1), and hence no evidence for the Wahlund effect. Italy also had the largest *N*_e_ estimate and Anatolia the smallest, consistent with the very small modern population in Anatolia (Masseti and Mertzanidou, 2008; De Marinis and Masseti, 2009). However, compared with other species, even the results for Italy are low. A European red deer phylogeography study reported *H*_e_ to vary from 0.33 to 0.73 and AR from 2.52 to 6.32 in red deer among 13 loci (Zachos *et al.*, 2016). A comparable roe deer study reported *H*_e_ to vary from 0.53 to 0.79 and alleles per locus to range from 3.4 to 6.5 among 11 loci (Randi *et al.*, 2004). Even among roe deer populations in the United Kingdom, heavily affected by introductions and founder events, there were 10 alleles per locus on average among 16 loci, and *H*_e_ ranged from 0.57 to 0.76 (Baker and Hoelzel, 2013). The very low levels of diversity suggest small effective population sizes.

Measures of population differentiation were also exceptional. With the exception of relatively low values between Portugal and Spain (*F*_ST_=0.031) and between England and Ireland (*F*_ST_=0.053), all other putative populations were highly differentiated for microsatellite DNA loci, with *F*_ST_ ranging from 0.278 to 0.815. This is much higher than seen among populations of red deer in Poland (*F*_ST_ ranged from 0.0 to 0.198; Feulner *et al.*, 2004), or the range seen among the fragmented and bottlenecked UK roe deer populations (0.05–0.31; Baker and Hoelzel, 2013). Although more restricted in geographic range than our study, other fallow deer studies (for example, for introduced populations in Tazmania; Webley *et al.*, 2007) also found relatively high levels of differentiation and low diversity within populations.

Both the low levels of diversity within regional populations and the high degree of differentiation between them is consistent with populations being founded by low numbers of individuals and affected by strong genetic drift. Examples can be seen among other mammal populations where differentiation between source and introduced populations has evolved over a relatively recent time frame (within 50–100 years; Maudet *et al.*, 2002; Baker and Hoelzel, 2013). However, the magnitude of differentiation is typically much smaller than the *F*_ST_ values seen here, even when the bottleneck events are very severe (for example, Lovatt and Hoelzel, 2014). As calculated in Lovatt and Hoelzel (2014), the Allendorf-Phelps model (Allendorf and Phelps, 1981) would predict an *F*_ST_ of 0.078 after a one-generation bottleneck of 7 individuals, close to the value observed for the reindeer (*Rangifer tarandus*) in that study (*F*_ST_=0.072). The magnitude of diversity among our study populations (*F*_ST_=0.278 to 0.815) might suggest prolonged or multiple bottleneck events, possibly linked to the tendency for fallow deer to slowly disperse from points of release (Fletcher, 2014) leading to small, localised and discontinuous populations. Furthermore, the historical and modern handling and distribution of fallow deer in parks is also relevant. Most specifically, this affects the English populations indicated in Table 1, some of which were established in parks during the medieval period (Sykes *et al.*, 2016). However, there are fewer apparent genetic divisions than park divisions in our sample set and they do not fully correspond, and hence the genetic structure may primarily reflect the original founder populations.

### Translocations

The impact of drift means that it is not always possible to track the sequence of events or source–founder population relationships, though some were evident. For example, the clear clustering of northern populations based on microsatellites (Figures 4 and 5) and mtDNA (Figure 6) is consistent with introduction records. The modern English fallow deer descends from an early medieval introduction (Sykes *et al.*, 2016) and these populations in the United Kingdom apparently subsequently founded those in Sweden (in the sixteenth century), Canada (in 1895; Chapman and Chapman, 1980; Obee, 1987) and likely Ireland (with introductions starting in the late medieval period; Whitehead, 1964). A connection between the assignment classes in the United Kingdom and each of Ireland, Sweden and Canada can be seen from the Structure analyses (Figure 4). The founding of Irish populations from the United Kingdom is also suggested from the BayesAss analysis (Supplementary Figure S3).

The pattern of diversity within the United Kingdom is complex, with HWE deviations suggesting a Wahlund effect, and Structure supporting multiple clusters. Borovali (1986) claims Western European deer descended from Anatolia, where animals were captured and transported up until the fifteenth century (see Masseti, 1998). A Turkish origin of Northern European populations would be consistent with the finding that these populations lack the 21 bp mtDNA insertion present in 88% of modern Italian and Spanish individuals. At the same time, microsatellite *F*_ST_ comparisons (Figure 3) and ordination graphs (Figure 5 and Supplementary Figure S2) suggest that in general the northern populations may have had significant input from the Italian population, especially for the United Kingdom. It is also possible that missing data, or a poor correspondence between the historical and modern population in Turkey, is complicating our interpretation of the pattern of founder events into northern populations.

Other introduced populations clearly include Rhodes that has a fixed, unique mtDNA indel distinguishing it from the other populations. There is no doubt that the current Rhodian population is unique and should be preserved in line with current IUCN (International Union for Conservation of Nature) recommendations (Masseti and Mertzanidou, 2008); however, it is clear that this population’s previously suggested Turkish ancestry (see Masseti, 2007; Masseti *et al.*, 2008) is more complex than previously thought. Our study provides support for input from Italian stock that would be consistent with claims that reintroductions took place on Rhodes during a period of the island’s Italian occupation (1912–1947) (Chapman and Chapman, 1980).

### Integration of anthropogenic and natural processes

All analyses based on the subset of data representing the traditional three refugial areas identified for a broad range of taxa (see Hewitt, 2000), the Iberian peninsula, the Italian peninsula and Anatolia, consistently showed strong differentiation, little or no internal structuring and no evidence for directional gene flow among them. We note that our approach in this study was to test a specific hypothesis about putative refugial populations in the context of known archaeological records (identifying translocations in central and northern Europe) and the geographic structure of southern Europe where three peninsulas define potential refugia. Although consistent with expectations, data showing distinct populations based on assignment or summary statistics can be interpreted in multiple ways. For example, the potential effects of strong founder events may show a similar pattern to differentiation in allopatry over time. However, ABC modelling allows us to further test whether all three putative refugial populations split at about the same time (as expected if they were separated when forced into refugia), compared with scenarios that would be more consistent with translocations. The results suggest that the best explanation was a split among these three southern populations in the late Pleistocene or early Holocene. This was clearest when the biparental microsatellite DNA markers were used on their own. The same pattern was supported when mtDNA was included, but the inference was weaker (Table 3 and Supplementary Figure S4), unexpected as the combination of different marker types generally reduces error in ABC analyses (see simulation studies in Cornuet *et al.*, 2010), but possibly because of factors such as incomplete lineage sorting or introgression in the mtDNA lineage. The timing could be consistent with very early translocation events, but could also be consistent with the establishment of refugia during the most recent glacial period (~110–12 Ka), as proposed for various other species (Warmuth *et al.*, 2011, Palkopoulou *et al.*, 2013, Weir *et al.*, 2016). At the same time, earlier dates have been suggested for establishing refugial populations for various species in Europe (see Hewitt, 2000 for a review, but compare with discussion of faster mutation rates than proposed in the earlier studies, for example, Ho *et al.*, 2007 that then dates some of these events to the recent glacial period).

Various studies suggest that an Anatolian refuge contributed significantly towards the recolonisation of many other European species (for review see Bilgin, 2011). Although the lack of available modern samples representing the original population complicates inference, the data do not support the assertion that all European fallow deer originate from Anatolia (see Sykes *et al.*, 2011). Genetic signals for additional refugia from Italy and Iberia were suggested most clearly from the microsatellite data (DIYABC, Structure, factorial correspondence analysis), whereas the mtDNA Network analysis was less clear with respect to the distinction between Italy and Iberia (though each are distinct from Anatolia), possibly because of incomplete lineage sorting or introgression. Some have suggested that Italy constituted its own fallow deer refugia (Masseti, 1996, 1999), but an argument for an Iberian refugia has not been previously made, as the archaeological appearance of fallow deer in Spain strictly coincided with the arrival of the Romans (Davis and MacKinnon, 2009) who began the widespread movement of this species across continental Europe (Pascal *et al.*, 2006).

It is possible that the lack of early Holocene records of fallow deer from archaeological sites in Iberia is because the culture associated with fallow deer developed later in that region. Alternatively, the Iberian population may represent an early human-mediated translocation from a now extinct refugial source. Candidate populations have been suggested in the Balkans (including Bulgaria and possibly Greece; Masseti, 1996, 1999). Our samples from central Europe, including Bulgaria, group with other putative translocated populations in the north (for both mtDNA and microsatellite DNA loci; see Figures 5 and 6). Any further translocations in the southern range should have resulted in some indication of admixture or substructure, but little was found (see Figure 4 and Supplementary Table S3). In fact, there is a clear contrast between northern and southern populations, such that the northern sites (including central Europe) are consistent with known founder events and translocations, whereas in the south most differentiation is between the three putative refugial areas. However, the origin of the Iberian lineage and the inclusive identification of refugia remain open questions. Ancient DNA, especially from the Balkan regions, may help resolve this (and clarify the conservation status of this species, see Chakanya *et al.*, 2016).

The persistent signal for differentiated regional populations in the southern part of the range is of interest, and consistent with some other postglacial distributions in Europe. For example, although European hedgehogs (*Erinaceus* sp.) are known to be heavily affected by anthropogenic barriers such as roads (Becher and Griffiths, 1998), the wider pattern of genetic diversity continues to reflect the likely recolonisation path out of glacial refugia (Seddon *et al.*, 2001). For fallow deer, interacting with these underlying processes is a complex pattern of reintroduction into northern distributions (with some strong influence from the relatively diverse Italian population) and, consistent with known historical events, further reintroductions from populations in the United Kingdom into Canada, Ireland and Sweden, though disentangling the pattern of anthropogenic translocations is difficult using modern data alone.

## Conclusions

Collectively, the data presented have implications for the wider conservation of this species. Fallow deer are currently classified as least concern by the IUCN, with special consideration given to Turkish and Rhodian populations on the basis that they represent ancient indigenous stock (see Masseti and Mertzanidou, 2008). Clearly, conservation efforts should continue in these regions (especially given very low genetic diversity); however, it is also clear that conservation should extend to other European populations that are depauperate of variation and significantly differentiated from each other (potentially reflecting lineages of evolutionary importance). More broadly, our study illustrates that a signature reflecting postglacial refugia can still be detected despite extensive anthropogenic manipulation. At the same time, for at least some aspects of the genetic structure, the timing and pattern of population divisions is predicted by the historical data on known patterns of translocation.

## Data accessibility

DNA sequences: Genbank accessions KY564399–KY564432; Microsatellite DNA genotypes: Dryad Digital Repository http://dx.doi.org/10.5061/dryad.d2g8v.

## Figures and Tables

**Table 1:** Microsatellite diversity statistics for fallow deer samples at each location

*Country*	*Locations*	*Map no.*	N *samples*	A	*AR*	F_*IS*_	H_*o*_	H_*e*_	*P-value*
*Spain*									
Central	North Madrid and Toledo	13	15	1.7	1.51	0.21	0.16	0.20	0.75
South west	Cadiz Huelva and Jaen	12	25	2.3	1.75	0.13	0.20	0.23	0.83
North	Aiguamolls and Rialp	14	15	1.6	1.47	−0.21	0.21	0.18	0.91
	Asturias	11	15	1.8	1.60	0.15	0.15	0.17	0.22
									
*Portugal*									
East central	Castelo Branco	10	13	1.7	1.61	0.15	0.19	0.23	0.89
									
*Italy*									
North	Grosetto	18	15	2.3	2.21	0.04	0.42	0.43	0.25
	Siena	19	14	2.6	2.42	0.08	0.45	0.48	0.63
	San Rossore	20	14	2.2	2.05	0.11	0.37	0.41	0.19
									
*England*									
Central south	Essex	8	15	2	1.88	−0.03	0.34	0.33	0.29
East	Oxfordshire (P)	6	9	2.2	2.13	0.28	0.25	0.33	0.04
	Cambridge and Bedfordshire	7	15	2	1.90	−0.04	0.35	0.34	0.08
North	Shropshire (P) and Cheshire (P)	4	9	2.2	1.76	−0.12	0.29	0.26	0.18
	Lincolnshire (P)	5	15	2.1	1.90	0.01	0.30	0.31	0.18
	Norfolk (P)	9	24	2.2	2.00	0.08	0.35	0.38	0.07
									
*Ireland*									
N central	Roscommon, Galway and Clare	1	13	2.1	1.97	0.19	0.25	0.31	0.64
East	Wicklow	3	13	1.8	1.62	0.29	0.16	0.22	0.08
South	Waterford, Kilkenny and Tipperary	2	10	2	1.94	0.29	0.26	0.36	0.45
									
*Sweden*									
South	Kristianstad, Maltesholm	15	15	2.1	1.97	−0.03	0.35	0.34	0.36
									
*Hungary*									
East	Gyula	17	7	2.2	2.10	−0.14	0.34	0.30	0.93
West	Labod	16	7	2.1	2.07	−0.06	0.39	0.37	0.52
									
*Canada*									
South	British Colombia, Sidney Island	Not shown	24	1.6	1.46	−0.09	0.15	0.14	0.83
									
*Turkey*									
South west	Antalya, Düzlerçami (P)	23	24	1.7	1.53	−0.22	0.22	0.18	<0.01
Rhodes		24	24	2.7	2.12	0.23	0.26	0.34	0.01
									
*Bulgaria*									
South	Eastern Rhodopes	22	14	1.7	1.62	0.10	0.19	0.21	0.95
	Totals and averages		364	2.038	1.86	0.06	0.28	0.29	

Abbreviations: A, number of alleles; AR, allelic richness; *F*_IS_, inbreeding coefficient; *H*_e_, expected heterozygosity; *H*_o_, observed heterozygosity.

*P*-values are indicated for multilocus Hardy–Weinberg equilibrium tested against the null hypothesis.

Samples originating from animals in enclosed parks are denoted parenthetically by ‘P’ after location names.

**Table 2:** MtDNA diversity metrics

	*Map no.*	N	k	h	π	k/N
Spain	11, 12, 13	19	4	0.456	0.00069	0.211
Portugal	10	17	3	0.647	0.00507	0.177
Italy	18, 19, 20	30	7	0.731	0.01029	0.233
England	4, 5, 6, 7, 8, 9	57	15	0.902	0.0031	0.263
Ireland	1, 2, 3	16	4	0.442	0.00068	0.25
Hungary	16, 17	13	3	0.513	0.0008	0.231
Turkey	23	20	2	0.1	0.00028	0.1
Bulgaria	22	11	1	0	0	0.091
Canada	Not shown	3	1	–	–	–
Rhodes	24	4	2	–	–	–
Sweden	15	7	1	–	–	–
Slovenia	25	3	2	–	–	–

Abbreviation: MtDNA, mitochondrial DNA.

Sample size (*N*), haplotype number (*k*), haplotype diversity (*h*), gene diversity (*π*) and haplotypes per individual (*k*/*N*) are shown.

Diversity measures are only given when *N*>10.

**Table 3:** Posterior parameter estimates from ABC analyses for scenario 1 (see Figure 2), where *N*1, *N*2 and *N*3 are effective population size estimates for the extant populations, Tb is the estimated date of lineage splitting and *N*_a_ is the ancestral effective population size

*Parameter*	*Msat only*	*Msat and mtDNA*
*N*1 (Iberia)	1060 (152–4700)	1120 (195–4150)
*N*2 (Italy)	4190 (955–8880)	2650 (525–7610)
*N*3 (Turkey)	1800 (281–6090)	422 (67–1860)
Tb (YBP)	12 700 (1850–38 000)	14 700 (3190–42 500)
*N*_a_	987 (39–3910)	238 (16–910)

Abbreviations: ABC, approximate Bayesian computation; mtDNA, mitochondrial DNA.

We assume a generation time of 1, but see text for alternative interpretations.
