# Supplementary material for: Strong population structure in a species manipulated by humans since the Neolithic: the European fallow deer (Dama dama dama)
Source: Heredity (Edinb). 2017 Mar 29;119(1):16–26. doi: 10.1038/hdy.2017.11 (PMC5520134; doi:10.1038/hdy.2017.11)

**Supplementary File**

Table S1: Microsatellite loci, their primer sequences and optimised amplification conditions where T_a_ is annealing temperature, t is for a touch-down protocol, A is for number of alleles and ref is the publication source of the microsatellite. PCR was carried out using Qiagen™ Multiplex kit (Qiagen) in a total volume of 5 µl containing 0.2 µM of each primer (forward primer was labelled with a fluorophor) and 0.5 µl of template DNA. PCR reactions were carried out under the following conditions; 15 min at 95°C, followed by 35 cycles of 30 sec at 94°C denaturation step, 90 sec annealing step and 1 minute at 72°C elongation step. A final extension step was carried out for 30 min at 60°C. Annealing temperatures were optimised for each of the primer sets (see Table S1). Genotyping was carried out on a 3730 ABI DNA Analyser (DBS Genomics, Durham, UK) and data was size-scored using Peak Scanner™ v. 1.0 (Applied Biosystems, Foster City, California). Refs: 1: Poetsch et al. 2001, 2: Buchanan et al. 1994, 3: Hawkins et al. 1995, 4: Crawford et al. 1995, 5: Moore et al. 1994, 6: Bishop et al. 1994, 7: Barendse et al. 1994, 8: Kemp et al. 1995, 9: Ihara et al. 2004.

|  | **Locus** | **Primer sequence (5’-3’)** | **Dye** | **T_a_ [°C]** | **A** | **Ref** |
| --- | --- | --- | --- | --- | --- | --- |
| PCR 1 | OarFCB48 | GACTCTAGAGGATCGCAAAGAACCAG | FAM | 60 | 5 | 2 |
|  |  | GAGTTAGTACAAGGATGACAAAGAGG |  |  |  |  |
|  | BM4505 | ATCTTCACTTGGGATGCAGG | TAM |  | 4 | 6 |
|  |  | TTATCTTGGCTTCTGGGTGC |  |  |  |  |
|  | ETH2 | CCCACAGGTGCTGGCATGGCC | HEX |  | 7 | 3 |
|  |  | CCATGGGATTTGCCCTGCTAGCT |  |  |  |  |
| PCR 2 | MAF70 | CACGGAGTCACAAAGAGTCAGACC | TAM | 66-60_t_ | 4 | 4 |
|  |  | GCAGGACTCTACGGGGCCTTTGC |  |  |  |  |
| PCR 3 | ILST30 | CTGCAGTTCTGCATATGTGG | HEX | 54 | 3 | 8 |
|  |  | CTTAGACAACAGGGGTTTGG |  |  |  |  |
| PCR 4 | HAUT27 | TTTTATGTTCATTTTTTGACTGG | TAM | 54 | 4 | 9 |
|  |  | AACTGCTGAAATCTCCATCTTA |  |  |  |  |
| PCR 5 | CSSM014 | AAATGACCTCTCAATGGAAGCTTG | TAM | 57 | 4 | 5 |
|  |  | AATTCTGGCACTTAATAGGATTCA |  |  |  |  |
|  | NVHRT21 | GCAGCGGAGAGGAACAAAAG | TAM |  | 4 | 1 |
|  |  | GGGGAGGAGCAGGGAAATC |  |  |  |  |
|  | RT30 | CACTTGGCTTTTGGACTTA | TAM |  | 4 | 1 |
|  |  | CTGGTGTATGTATGCACACT |  |  |  |  |
|  | TGLA127 | CAATTGTGTGGTAGTTTGGACATTC | HEX |  | 5 | 7 |
|  |  | CACTATTGCAAAGGACCTCCAATT |  |  |  |  |

Table S2: List of alleles observed within each fallow deer population and locus.

|  | OarFCB48 | MAF70 | BM4505 | HAUT27 | CSSM014 | NVHRT21 | RT30 | ETH2 | ILST30 | TGLA127 |
| --- | --- | --- | --- | --- | --- | --- | --- | --- | --- | --- |
| Spain | 153 163 167 | 110 126 | 252 254 264 | 136 140 142 | 146 | 176 | 201 203 205 | 175 179 | 143 145 149 | 160 170 174 176 |
| Portugal | 153 163 167 | 110 126 | 252 | 136 140 | 146 | 176 | 201 203 | 175 179 | 149 | 170 174 176 |
| Italy | 153 163 167 | 110 126 | 252 254 264 | 136 140 142 | 142 146 | 176 178 180 | 201 205 | 179 191 183 | 143 145 149 | 170 174 |
| England | 153 163 167 | 110 126 | 252 254 264 266 | 140 142 146 | 142 146 152 154 | 176 178 | 201 205 | 175 181 183 | 143 145 | 160 170 174 |
| Ireland | 153 | 110 126 | 252 254 264 | 140 142 146 | 142 154 146 | 176 | 201 205 | 175 181 | 143 145 | 160 170 174 |
| Sweden | 153 | 110 126 | 252 254 264 | 140 142 | 142 146 154 | 176 | 201 205 | 175 179 181 | 143 145 | 160 170 |
| Hungary | 153 163 165 167 | 110 126 | 252 254 264 | 140 142 | 142 146 154 | 176 178 | 201 | 179 181 191 | 143 145 | 160 174 |
| Canada | 153 | 110 126 | 252 264 | 136 142 | 142 146 154 | 176 | 201 | 181 | 143 145 | 174 |
| Turkey | 153 | 110 126 | 264 | 140 | 142 146 | 170 178 | 201 203 205 | 179 181 | 149 | 170 172 |
| Rhodes | 153 165 167 169 | 110 112 126 | 252 | 136 140 | 142 146 | 176 178 | 199 201 203 | 175 179 181 199 | 143 145 149 | 170 172 174 176 |
| Bulgaria | 153 163 | 110 126 | 252 264 | 142 | 142 152 | 176 | 143 145 | 181 | 143 145 | 174 |

Table S3: Pairwise microsatellite DNA F_ST_ values; all highly significant (p<0.00001) except for value in bold.

|  | Spain | Portugal | Italy | Turkey | Rhodes | Bulgaria | Hungary | England | Ireland | Sweden |
| --- | --- | --- | --- | --- | --- | --- | --- | --- | --- | --- |
| Spain |  |  |  |  |  |  |  |  |  |  |
| Portugal | **0.031** |  |  |  |  |  |  |  |  |  |
| Italy | 0.533 | 0.414 |  |  |  |  |  |  |  |  |
| Turkey | 0.733 | 0.722 | 0.515 |  |  |  |  |  |  |  |
| Rhodes | 0.675 | 0.593 | 0.368 | 0.652 |  |  |  |  |  |  |
| Bulgaria | 0.745 | 0.701 | 0.403 | 0.726 | 0.655 |  |  |  |  |  |
| Hungary | 0.625 | 0.512 | 0.296 | 0.697 | 0.536 | 0.287 |  |  |  |  |
| England | 0.629 | 0.557 | 0.278 | 0.613 | 0.563 | 0.228 | 0.265 |  |  |  |
| Ireland | 0.682 | 0.600 | 0.283 | 0.658 | 0.556 | 0.330 | 0.371 | 0.053 |  |  |
| Sweden | 0.671 | 0.572 | 0.254 | 0.644 | 0.483 | 0.378 | 0.347 | 0.264 | 0.280 |  |
| Canada | 0.747 | 0.742 | 0.362 | 0.815 | 0.647 | 0.492 | 0.457 | 0.262 | 0.280 | 0.430 |

Table S4: Theta (4Neu) and directional migration estimates given as *Nm* from Migrate, and proportion migrant estimates from BayesAss.

| **Parameter** | ***Migrate* (95% conf. Limits)** | ***BayesAss* (s.d.)** |
| --- | --- | --- |
| θ – Iberia | 1.07 (0.0-2.8) | - |
| θ – Italy | 2.00 (0.0-4.0) | - |
| θ – Turkey | 0.40 (0.0-2.0) | - |
| Italy͢→Iberia | 1.109 (0.0-4.4) | 0.0073 (0.0056) |
|  |  |  |
| Iberia→Italy | 1.741 (0.0-5.6) | 0.0113 (0.0096) |
|  |  |  |
|  |  |  |
| Turkey→Iberia | 0.804 (0.0-4.0) | 0.0043 (0.0042) |
| Iberia→Turkey | 0.786 (0.0-4.0) | 0.0123 (0.0118) |
|  |  |  |
| Turkey→Italy | 1.961 (0.0-5.6) | 0.0074 (0.0072) |
| Italy→Turkey | 0.793 (0.0-4.0) | 0.0124 (0.0119) |

Table S5: mtDNA control region haplotype list

| Haplotype number | No. of individuals | Haplotype by country |
| --- | --- | --- |
| Haplotype 1 | 1 | Turkey |
| Haplotype 2 | 19 | Turkey |
| Haplotype 3 | 33 | Ireland (12), England (9), Sweden (7), Slovenia (1), Portugal (4) |
| Haplotype 4 | 3 | Ireland (2), England (1) |
| Haplotype 5 | 35 | Ireland (1), England (9), Hungary (9), Germany (1), Bulgaria (11), Italy, Piedmont (4) |
| Haplotype 6 | 1 | Ireland (1) |
| Haplotype 7 | 4 | England (3), Germany (1) |
| Haplotype 8 | 9 | England (9) |
| Haplotype 9 | 16 | Ireland (1), England (8), Hungary (2), Canada (3), Slovenia (2) |
| Haplotype 10 | 4 | England (4) |
| Haplotype 11 | 7 | England (5), Germany (2) |
| Haplotype 12 | 1 | England (1) |
| Haplotype 13 | 2 | England (2) |
| Haplotype 14 | 2 | England (2) |
| Haplotype 15 | 1 | England (1) |
| Haplotype 16 | 1 | England (1) |
| Haplotype 17 | 4 | England (1), Hungary (2), Germany (1) |
| Haplotype 18 | 2 | Germany (2) |
| Haplotype 19 | 1 | Germany (1) |
| Haplotype 20 | 2 | Germany (2) |
| Haplotype 21 | 1 | Germany (1) |
| Haplotype 22 | 1 | Germany (1) |
| Haplotype 23 | 2 | Germany (1), Italy (1) |
| Haplotype 24 | 4 | Rhodes (4) |
| Haplotype 25 | 1 | Italy (1) |
| Haplotype 26 | 12 | Italy (12) |
| Haplotype 27 | 10 | Italy (10) |
| Haplotype 28 | 1 | Italy (1) |
| Haplotype 29 | 1 | Italy (1) |
| Haplotype 30 | 23 | Portugal (9), Spain (14) |
| Haplotype 31 | 4 | Portugal (4) |
| Haplotype 32 | 2 | Spain (2) |
| Haplotype 33 | 1 | Spain (1) |
| Haplotype 34 | 2 | Spain (2) |

Table S6: Pairwise mtDNA FST values. ns = no significance; *p<0.05; **p<0.01; ***p<0.00001

|  | Spain | Portugal | Italy | Turkey | Bulgaria | Hungary | England |
| --- | --- | --- | --- | --- | --- | --- | --- |
| Spain |  |  |  |  |  |  |  |
| Portugal | 0.211*** |  |  |  |  |  |  |
| Italy | 0.253*** | 0.133** |  |  |  |  |  |
| Turkey | 0.989*** | 0.790*** | 0.763*** |  |  |  |  |
| Bulgaria | 0.990*** | 0.712*** | 0.708*** | 0.982*** |  |  |  |
| Hungary | 0.983*** | 0.719*** | 0.713*** | 0.954*** | 0.065ns |  |  |
| England | 0.943*** | 0.795*** | 0.790*** | 0.780*** | 0.152*** | 0.135*** |  |
| Ireland | 0.980*** | 0.730*** | 0.723*** | 0.936*** | 0.656*** | 0.556*** | 0.098ns |

Table S7: ABC simulation 1 parameter comparisons for runs based on microsatellite DNA loci only, and for

combined microsatellite DNA and mtDNA data. Simulation mean and standard deviation based on

1000 replicates. * indicates significant difference at the 0.05 level.

| **Parameter** | **Observed** | **Mean ± s.d.** | **proportion (sim < obs)** |
| --- | --- | --- | --- |
| # alleles Iberia (msat only) | 2.5 | 2.466 ± 0.915 | 0.597 |
| # alleles Italy (msat only) | 2.7 | 4.221 ± 0.977 | 0.052 |
| # alleles Turkey (msat only) | 1.7 | 2.743 ± 0.859 | 0.079 |
| genic div. Iberia (msat only) | 0.208 | 0.245 ± 0.137 | 0.410 |
| genic div. Italy (msat only) | 0.460 | 0.514 ± 0.114 | 0.269 |
| genic div. Turkey (msat only) | 0.182 | 0.341 ± 0.141 | 0.121 |
| Fst Iberia v. Italy (msat only) | 0.512 | 0.521 ± 0.136 | 0.462 |
| Fst Iberia v. Turkey (msat only) | 0.685 | 0.630 ± 0.150 | 0.653 |
| Fst Italy v. Turkey (msat only) | 0.463 | 0.413 ± 0.119 | 0.673 |
| # alleles Iberia (msat & mtDNA) | 1.8 | 2.669 ± 0.675 | 0.050* |
| #alleles Italy (msat & mtDNA) | 2.7 | 3.619 ± 0.699 | 0.111 |
| # alleles Turkey (msat & mtDNA) | 1.7 | 1.863 ± 0.517 | 0.690 |
| genic div. Iberia (msat & mtDNA) | 0.214 | 0.352 ± 0.122 | 0.095 |
| genic div. Italy (msat & mtDNA) | 0.480 | 0.523 ± 0.097 | 0.410 |
| genic div. Turkey (msat & mtDNA) | 0.182 | 0.191 ± 0.110 | 0.761 |
| Fst Iberia v. Italy (msat & mtDNA) | 0.457 | 0.462 ± 0.110 | 0.555 |
| Fst Iberia v. Turkey (msat & mtDNA) | 0.694 | 0.658 ± 0.109 | 0.430 |
| Fst Italy v. Turkey (msat & mtDNA) | 0.485 | 0.568 ± 0.103 | 0.039* |

Figure S1: Posterior probability of the data (Ln [P(D|K)]) and values of ΔK (Evanno *et al.* 2005) as a function of K (number of clusters), associated with the results shown in Figure 2.


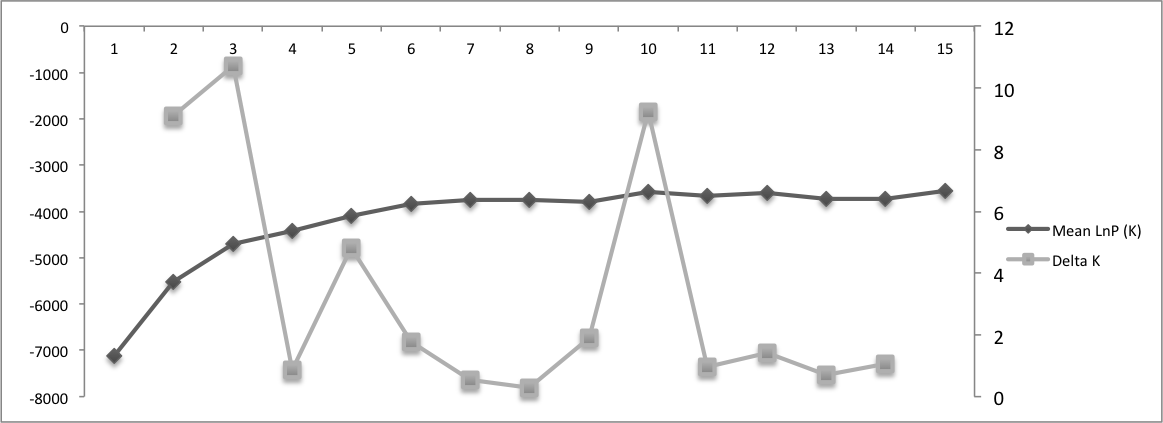


Figure S2: **Principal component analysis based on genetic distances of fallow deer.**

Figure S3: Circos plots from the BayesAss analyses for the three southern putative refugial populations (a) and all populations (b). Directional gene flow is indicated by lines connecting one region to another.


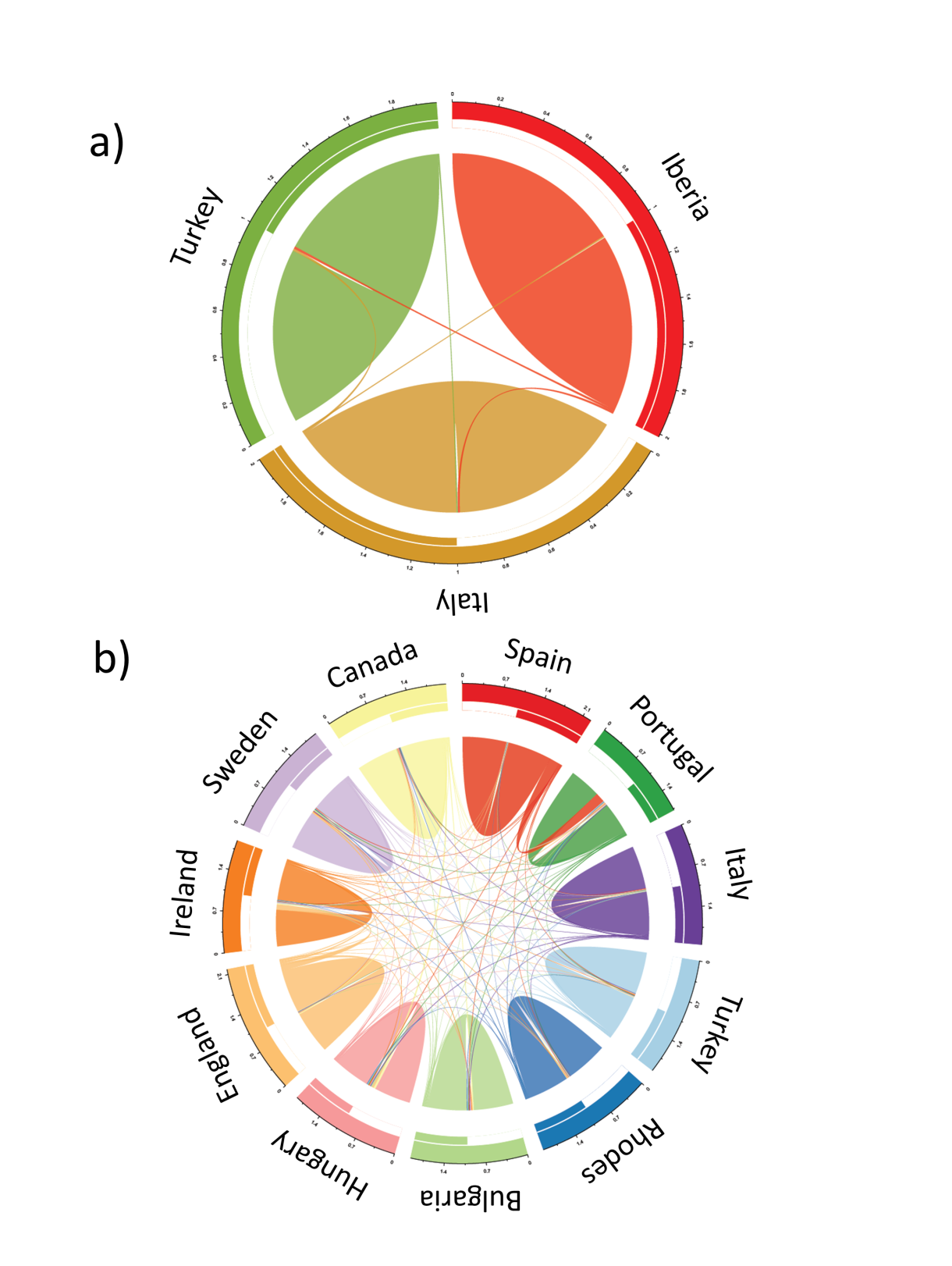


Figure S4: ABC direct and logistic regressions comparing the seven scenarios (see Figure 6). a) microstallite DNA loci only, b) microsatellite DNA loci with mtDNA locus.

a)


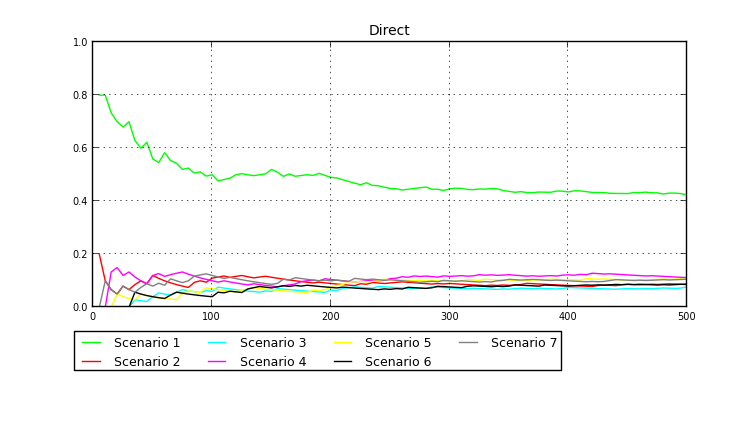


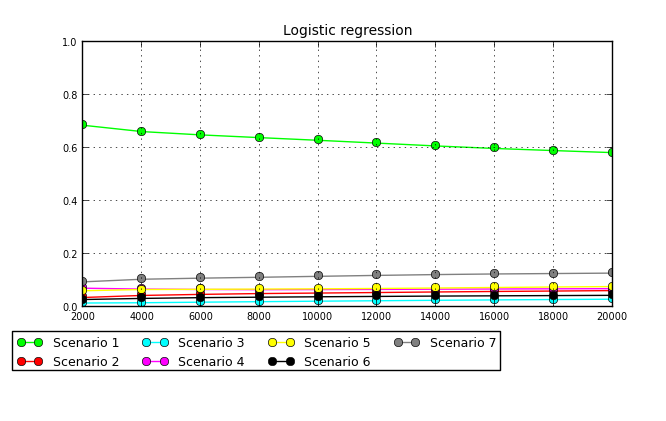


b)


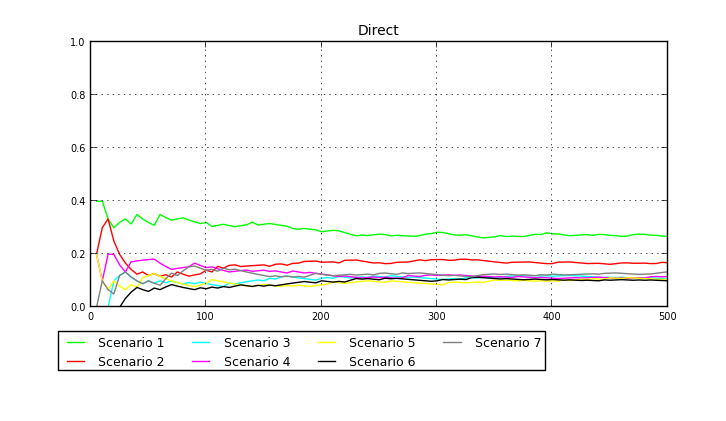


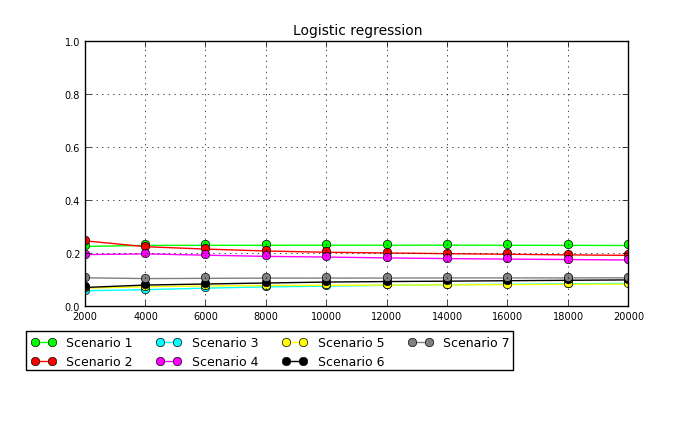


Figure S5: Scenario 1 PCA analysis of posterior (closed green circles) and prior (open green circles) distributions compared to the observed values (yellow circle) for the microsatellite only dataset (a) and for microsatellites and mtDNA combined (b).

a)


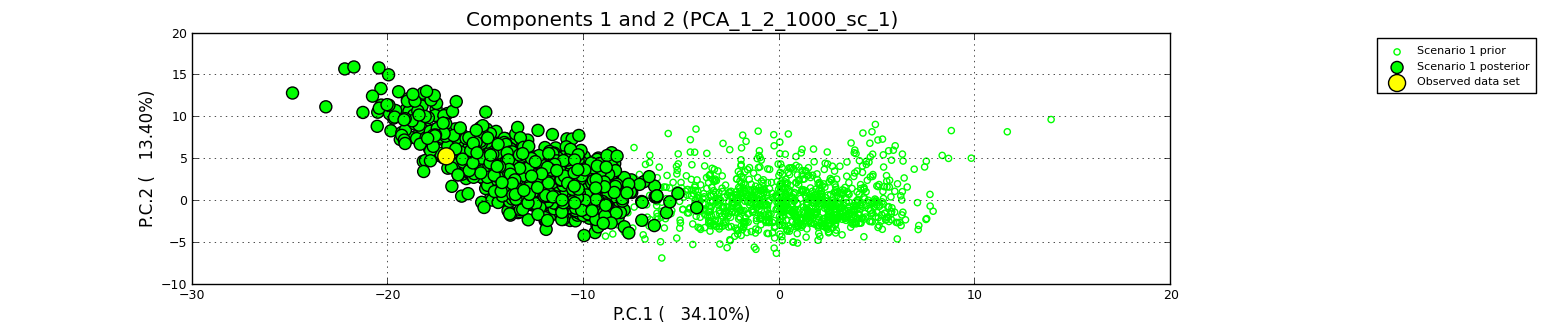


b)


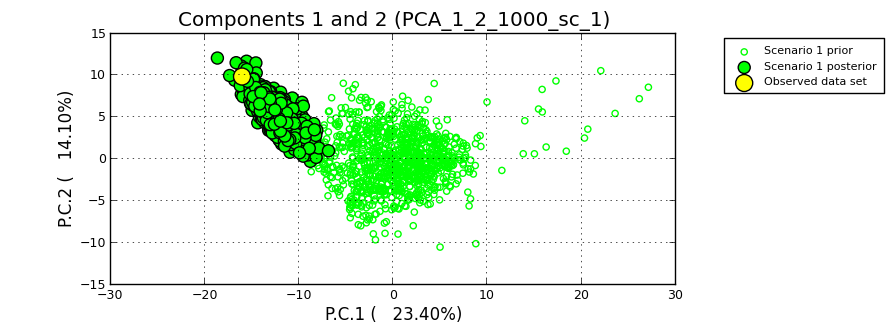

Supplement: Supplementary Material [file hdy201711x1.docx]
